# Evaluation of Antioxidant, Immunomodulatory Activities, and Safety of Ethanol Extract and Fractions of *Gongronema latifolium* Fruit

**DOI:** 10.1155/2014/695272

**Published:** 2014-10-28

**Authors:** Amanze Agwaramgbo, Emmanuel Emeka Ilodigwe, Daniel Lotanna Ajaghaku, Maureen Ugochukwu Onuorah, Sonne Ikechukwu Mbagwu

**Affiliations:** Department of Pharmacology and Toxicology, Faculty of Pharmaceutical Sciences, Nnamdi Azikiwe University, Awka, Anambra State 420281, Nigeria

## Abstract

*Gongronema latifolium* fruit has wide application in ethnomedicine, especially in maintaining healthy living and general body healing. We therefore investigated the antioxidant, immunomodulatory activities, and safety of its ethanol extract and fractions. The *in vitro* antioxidant activities of the extract and fractions were determined using 2,2-diphenyl-1-picrylhydrazyl (DPPH) test while *in vivo* activities were determined using carbon tetrachloride (CCL_4_) induced oxidative stress. Cell and humoral mediated immune responses were also evaluated together with toxicity studies. The extract, ethyl acetate, and methanol fractions showed inhibition of DPPH radical with IC_50_s 120, 90, and 60 *μ*g/mL, respectively. Methanol fraction at 200 mg/kg produced significant (*P* < 0.05) inhibition of lipid peroxidation (MDA conc. 1.2 *μ*mol/L) compared to control (2.8 *μ*mol/L). Both ethyl acetate and methanol fractions at 200 mg/kg produced significant (*P* < 0.05) phagocytic index of 0.021 and 0.025, respectively, compared with control (0.01). Significant (*P* < 0.05) elevations of white blood cells, aspartate aminotransferase, alanine aminotransferase, and alkaline phosphatase were noticed on the 91st day at higher doses. Generally, this study justified the traditional use of *G. latifolium* fruit for general body healing and maintenance of healthy living. Long term administration is safe on the haematological and biochemical systems especially at lower doses and its toxicity at higher doses is reversible.

## 1. Introduction

The human environment together with the biological processes contributes significantly to free radical production [1]. When these free radicals overwhelm the antioxidant defense mechanisms, oxidative stress sets in with its deleterious effects that lead to cell injury, degenerative diseases, and compromised immune system [2]. Antioxidants and immune boosting therapy have become very popular in the treatment of many disease conditions [3]. However, in many rural communities, antioxidant and immune boosting drugs are usually expensive, inadequately accessible, and usually associated with side effects. High reliance on natural products for health care maintenance is therefore very common in many communities and has led to increased search for readily available, cheap, and safe natural products with antioxidant and immune boosting properties.

*Gongronema latifolium* Benth (Asclepiadaceae) is a climber that is widely distributed in tropical Africa. It is commonly known as utazi in South Eastern Nigeria while in Ghana, Senegal, and Sierra Leone, it is called akan-asantes, gasub, and ndondo-polole, respectively [4]. Its leaves are used as leaf vegetable and spice in traditional Nigerian dishes [5]. The ethanol leaf extract has been shown to normalize renal oxidative stress and lipid peroxidation in diabetic rats [6]. Also, the leaf extract has hepatoprotective effect against acetaminophen [7]. The hypoglycemic, hypolipidemic, anti-inflammatory, and antioxidant activities of the leaf extract have all been reported [8–10]. The chemical composition of the leaf extract shows it has abundance of essential amino acids and fatty acids [11]. In South Eastern Nigeria, the fruit is very popular as a remedy for diabetes and high blood pressure, for maintaining healthy living, and in general body healing, yet no scientific study has been done to evaluate these folkloric uses. This study evaluated the antioxidant, immunomodulatory activities and safety of the ethanol extract and fractions of* G. latifolium *fruit.

## 2. Materials and Methods

### 2.1. Plant Material

The fruits of* G. latifolium* were collected from Nsukka, Enugu State, Nigeria, in March, 2012 and authenticated by a taxonomist, Mr. Alfred Ozioko of Bioresource Development and Conservation Project, Nsukka, Enugu State, Nigeria. The fruits were subsequently cleaned, air-dried under room temperature for 20 days, and pulverized.

### 2.2. Chemicals

Analytical grades of *n*-hexane, chloroform, ethyl acetate, methanol, hydrogen peroxide, hydrochloric acid, ethanol, DPPH (1,1-diphenyl-2-picrylhydrazyl), epinephrine, trichloroacetic acid, thiobarbituric acid, ovalbumin, and tetanus toxoid were all obtained from Sigma Chemicals CO, USA. All laboratory reagents including distilled water were freshly prepared when required.

### 2.3. Extraction and Fractionation

About 3 kg of the pulverized fruit was cold-macerated in aqueous ethanol (70%) for one week. The resulting solution was filtered using Whatman filter paper. The filtrate was concentrated to dryness* in vacuo* using rotary evaporator at 40°C. The extract (116 g) was adsorbed on silica gel and eluted in succession with *n*-hexane, chloroform, ethyl acetate, and methanol. The eluent was filtered and concentrated* in vacuo* using rotary evaporator at 40°C to obtain the *n*-hexane, chloroform, ethyl acetate, and methanol fractions. All the extracts and fractions were stored in the refrigerator between 0 and 4°C until used.

### 2.4. Phytochemical Test

The phytochemical analyses of the fruit extract and fractions were carried out using standard methods [12, 13]. Qualitative determination of secondary metabolites was done.

### 2.5. Animals

Swiss male and female albino rats 130 ± 7 g and mice 30 ± 4 g (3 months old) were employed for the study. All the animals were obtained from the animal house of the Department of Pharmacology and Toxicology, Nnamdi Azikiwe University, Agulu campus. The animals were housed in standard laboratory conditions of 12 h light, room temperature, and 40–60% relative humidity and fed with rodent feed (Guinea Feeds Nigeria Ltd.). They were allowed free access to food and water.

Albino rats were used for the antioxidant studies while mice were used for immunomodulatory studies. In both studies, the animals were divided into 8 groups of 5 animals each. Groups 1 and 2 received ethanol extract while groups 3 and 4 received ethylactate fraction and groups 5 and 6 methanol fraction. The extracts and fractions were administered orally at the doses of 200 and 400 mg/kg. Groups 7 and 8 served as the control and received 200 mg/kg of standard (vitamin C or Noni capsule) and 10% tween 80, respectively.

For the chronic toxicity studies, eighty male rats were randomly divided into four groups (A, B, C, and D) of twenty rats per group. The extract was administered orally at doses 750, 1500, and 3000 mg/kg to groups A, B, and C once daily for 90 days. The control, group D, received 10 mL/kg of normal saline. Physical observation of the animals was done on daily basis while their weights were taken every week.

All animal experiments were conducted in accordance with NIH guide for the care and use of laboratory animals [14] and approved by the institution research ethical committee.

### 2.6. Acute Toxicity Study

Acute toxicity study was carried out according to OECD guideline for acute oral toxicity testing. A total of six mice and rats were subjected to limit test at 5000 mg/kg BW. Three animals each were administered this dose (p.o.) and observed for 48 hours for mortality or obvious toxic symptoms and thereafter for 14 days. This was followed by three additional animals each at the same dose. The LD_50_ was categorised based on this guideline [15].

### 2.7. Free Radical Scavenging Activity: DPPH Test

The DPPH test was evaluated using the method of Patel and Patel [16]. DPPH solution (0.6 Mmol) was freshly prepared using methanol as solvent and 0.5 mL of this solution was mixed with 0.5 mL of different dilutions (62.5, 125, 250, 500, and 1000 *μ*g/mL) of the extract and fractions. The final volume of the resulting solution was adjusted to 5 mL using methanol. The absorbance of the mixtures was measured at 520 nm after incubation in the dark for 30 minutes at room temperature. Ascorbic acid was used as standard.

The absorbance of the test substances and the negative control (0.5 mL of DPPH solution and 4.5 mL of methanol) was compared and the value used as parameter for the evaluation of antioxidant property. The free radical scavenging activity was obtained using the relationship shown below.

 DPPH scavenging activity = 100{(AC − AS)/AC}.
 AC = absorbance of negative control.
 AS = absorbance of Sample.

### 2.8. *In Vivo* Antioxidant Assay

The animals were anesthetized with light ether anaesthesia and blood samples (2 mL) were collected from the animals through retroorbital plexus into plain tubes for the pretreatment basal serum antioxidant assay. After the blood collection, the animals were treated with the extract and fractions once per day for 21 days. The animals were then given the acute oral dose of CCL_4_, 1.5 mL/kg BW. Blood samples were collected from the animals through retroorbital plexus 8 h after CCL_4_ administration. The serum was used for the determination of antioxidant enzyme activity (Catalase, SOD) and lipid peroxidation according to the method described by Ogbunugafor et al. [17]. Superoxide dismutase activity was determined by its ability to inhibit the autooxidation of epinephrine and catalase activity, by its ability to react with hydrogen peroxide while lipid peroxidation was quantified through formation of malondialdehyde- (MDA) thiobarbituric acid (TBA) complex.

### 2.9. Evaluation of Phagocytic Activity

The phagocytic index of the extract and fractions was determined using carbon clearance test. The animals were treated with the extract and fractions for 14 days. After 3 hrs posttreatment on the 14th day, the animals were given intravenous injection through the tail vain of 1 mL/100 g of Indian ink. Blood samples were drawn through retroorbital plexus into heparinised EDTA tubes at 0 and 15 minutes after injection. 25 *μ*L of each of the blood samples was mixed with 3 mL of sodium carbonate and optical density was measured spectrophotometrically at 650 nm. The phagocytic index (*K*) was calculated as follows [18]:

(1) $$\begin{array}{c} K=\frac{\text{in}{\text{OD}}_{1}-\text{in}{\text{OD}}_{2}}{t_{2}-t_{1}}, \end{array}$$

where OD_1_ and OD_2_ are optical densities at *t*_1_ and *t*_2_. *t*_1_ and *t*_2_ are time intervals at 0 and 15 minutes.

### 2.10. Delayed Type Hypersensitivity Response

This was evaluated using the method of Makare et al. [19]. On the 21st day of daily oral administration of the extract and fractions, the thickness of the right hind foot pad was measured using vernier calliper before subplanter injection of 0.1 mL of 20% sheep red blood cell (SRBC). Subplanter thickness was remeasured after 24 h and the difference between the pre- and postinjection was taken as the measure of delayed hypersensitivity response.

### 2.11. Blood Leukocyte Count

Following 14th day of daily treatment, blood samples were collected through retroorbital plexus into heparinised EDTA tubes and analysed for total (TLC) and differential leucocyte counts (DLC) by fixing blood smear and staining with Leishman's stain.

### 2.12. Immunization and Immunoglobulin Levels Determination

For the ovalbumin (OVA) and tetanus toxoid immunization, the animals were intraperitoneally injected with 10 *μ*g/mL ovalbumin or tetanus toxoid on the 5th and 14th days. Blood samples were collected through retroorbital plexus on the 14th and 21st days for determination of primary and secondary antibody responses, respectively. The antisera were separated and used for the estimation of IgG_1_ and IgG_2a_ using ELISA as described by Duddukuri et al. [20].

### 2.13. Subchronic Toxicological Studies

Five rats from each group were anaesthetized using light ether anaesthesia on the 31st, 61st, and 91st days of the study. Blood samples were collected through retroorbital puncture and heparinised whole blood samples were used for the estimation of haemoglobin (Hb), packed cell volume (PCV), red blood cell (RBC), and white blood cell (WBC) counts using the method described by Kelly [21]. For the estimation of serum liver enzymes, nonheparinised blood samples were allowed to coagulate and the clear serum separated by centrifuging at 2500 rpm for 10 minutes was then used for the analysis of alanine aminotransaminase (ALT) [22], aspartate aminotransaminase (AST) [22], and alkaline phosphatase (ALP) [23]. After the 90-day treatment studies, the remaining 5 rats in each group were returned to normal diet for 28 days and were used for assessing the reversibility or otherwise the toxic effects of the fruit extract on the haematological and biochemical parameters. Histopathological studies were done on liver isolates from different groups of the animals and at various times.

### 2.14. Statistical Analysis

The results were analysed using SPSS version 18 and presented as mean ± SEM. Mean comparison was determined using one-way ANOVA and turkey test. Differences between control and extract treated groups were considered significant at *P* < 0.05 and *P* < 0.01.

## 3. Results

### 3.1. Phytochemical Analysis

The phytochemical screening of the extract and fractions of* G. latifolium* fruit showed the presence of various chemical constituents (Table 1). Alkaloids, saponins, flavonoids, terpenoids, steroids, glycosides, and proteins were present in large amounts in the crude extract while flavonoids and proteins were found more in the ethyl acetate fraction. Saponins, flavonoids, proteins, and glycosides were abundant in the methanol fraction with moderate proportions of terpenoids, tannins, and carbohydrates. Ethanol extract yield from the powdered fruit was moderate (28.6%). The distribution of this extract into fractions of different solvent polarity was highest in methanol fraction followed by ethyl acetate and chloroform, respectively, with *n*-hexane having the least portion of the extract. See Table 1 for the yields.

### 3.2. *In Vitro* Antioxidant Activity: DPPH Test

**Figure 1:**
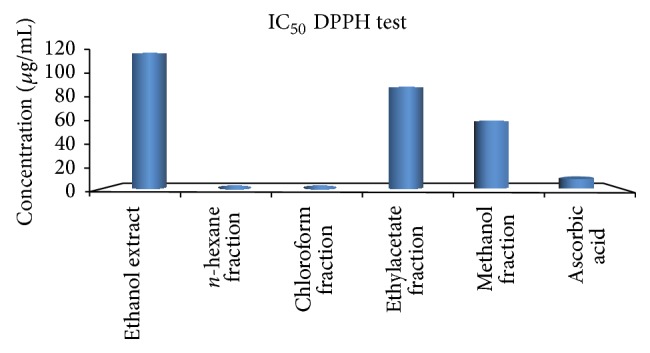
*In vitro* antioxidant activity of the ethanol extract and fractions of* G. latifolium* fruit using DPPH method.

The DPPH scavenging activity of methanol fraction was higher than that of ethyl acetate fraction and ethanol extract, respectively, while *n*-hexane and chloroform fractions did not produce inhibition up to 50% inhibition. These effects were however lower than that produced by ascorbic acid (Figure 1). Following the effects of these extract and fractions on DPPH, only the ethanol extract, ethyl acetate, and methanol fractions were selected for further antioxidant studies.

### 3.3. Acute Toxicity Study

The LD_50_ of the extract was above 5000 mg/kg BW for both animal species used. There were no observations of obvious toxic symptoms throughout the period of the study.

### 3.4. *In Vivo* Antioxidant Assay

**Figure 2:**
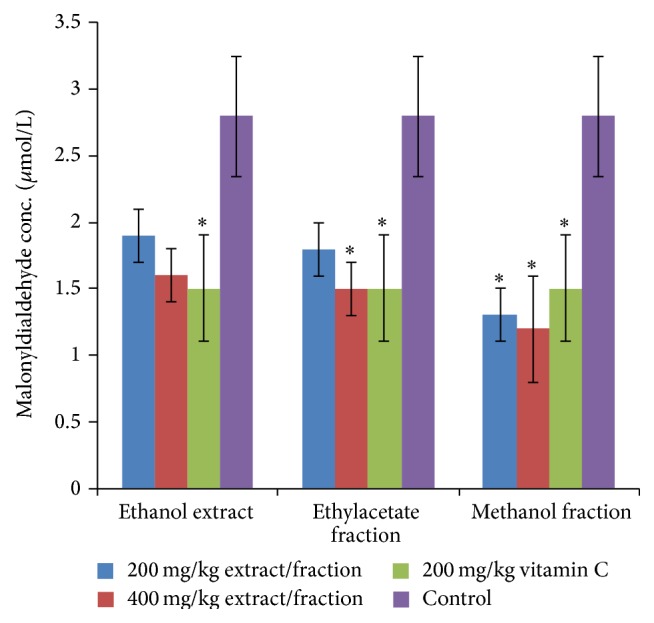
Effect of the extract and fractions of* G. latifolium* fruit on lipid peroxidation.

Ethyl acetate and methanol fractions produced significant (*P* < 0.05) increase in serum catalase activity that is higher than that of vitamin C (Table 2) while ethanol extract, ethyl acetate, and methanol fractions produced significant (*P* < 0.05) increase in serum superoxide dismutase enzyme just as vitamin C (Table 2). Methanol fraction at all doses and ethyl acetate fraction at 400 mg/kg produced significant (*P* < 0.05) reduction in lipid peroxidation compared with the control (Figure 2).

### 3.5. Phagocytic Index and Delayed Type Hypersensitivity Reaction

The extract at 400 mg/kg BW and the fractions at the tested doses increased clearance of carbon particles from the blood as indicated by significant (*P* < 0.05) increase in phagocytic index while, in the delayed type hypersensitivity reaction, the extract and fractions at 200 and 400 mg/kg BW produced significant increase in paw thickness when compared with negative control. Noni capsule (50 mg/kg) also showed significant (*P* < 0.05) phagocytic index and delayed type hypersensitivity reactions (Table 3).

### 3.6. Blood Leucocyte Count

The extract at 400 mg/kg and the fractions at all the doses produced significant elevation of total leukocytes just as the standard (Noni capsule); however only ethyl acetate fraction at 400 mg/kg showed differential significant (*P* < 0.05) elevation of neutrophils (Table 4).

### 3.7. Immunoglobulin Levels

The extract and fractions at all doses produced significant (*P* < 0.05) stimulation of IgG_1_ and IgG_2a_ just as the standard (Noni capsule) at 50 mg/kg BW against both ovalbumin and tetanus toxoid (Table 5). Greater response was observed following secondary encounter of these antigens as indicated by greater significant (*P* < 0.05) production of IgG_1_ and IgG_2a_ (Table 6).

### 3.8. Subchronic Toxicity Study

**Figure 3:**
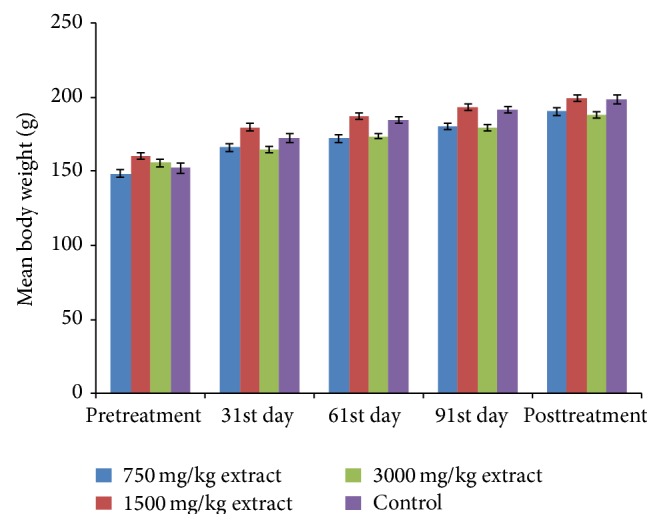
Effect of the extract on body weight.

**Figure 4:**
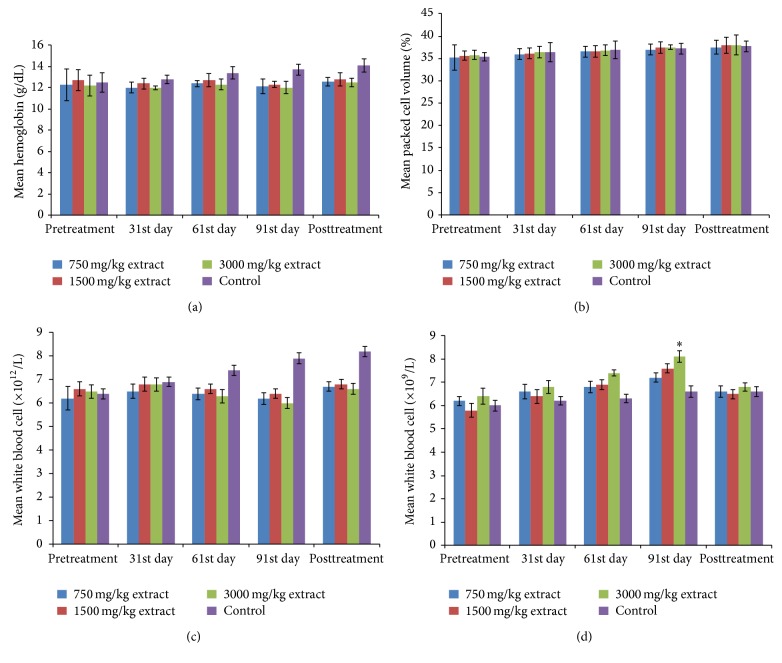
Effect of the extract on haematological parameters: (a) haemoglobin, (b) packed cell volume, (c) red blood cell, and (d) white blood cell.

**Figure 5:**
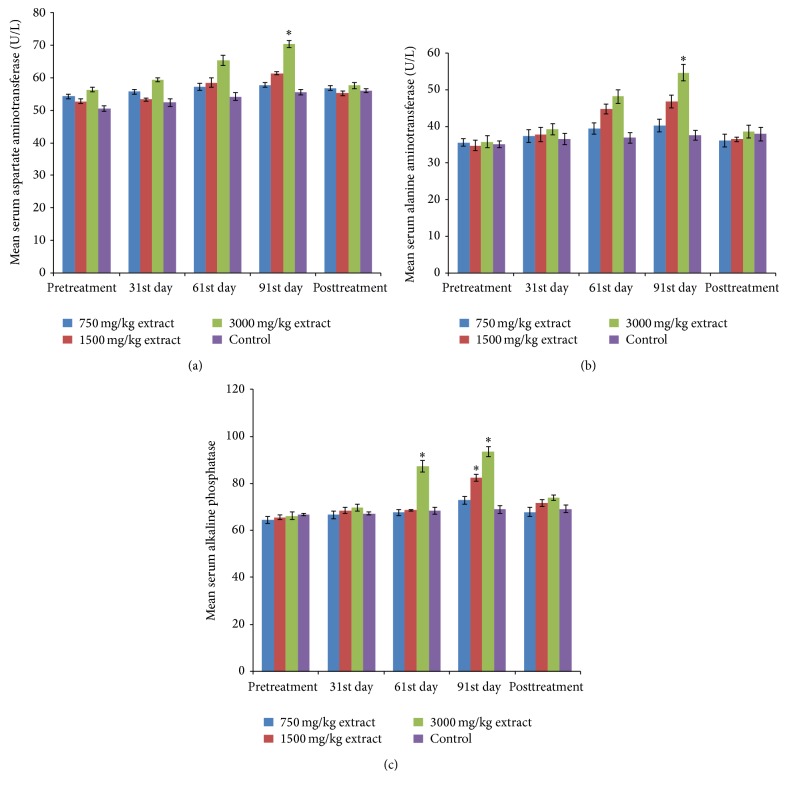
Effect of the extract on serum liver enzymes: (a) aspartate aminotransferase, (b) alanine aminotransferase, and (c) alkaline phosphatase.

**Figure 6:**
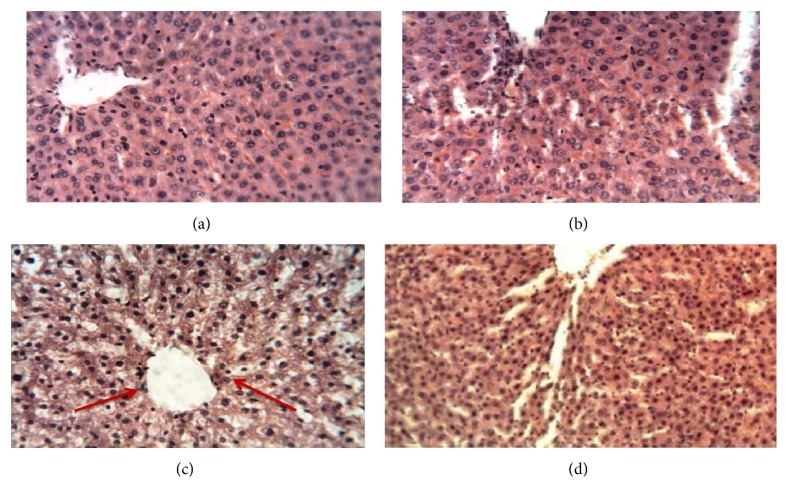
Liver histological photomicrograph, H&E staining, 40x: (a) control group on the 31st day, (b) 1500 mg/kg extract treated group on the 91st day, (c) 3000 mg/kg extract treated group on the 91st day—arrows showing vacuolar degenerations, and (d) liver photomicrograph for 3000 mg/kg after 28-day posttreatment.

No death was recorded throughout the chronic toxicity studies. The extract does not produce significant (*P* > 0.5) alteration of the body weights (Figure 3), PCV, Hb, and RBC counts (Figure 4). Significant (*P* < 0.05) elevation of WBC count (Figure 4(c)), AST, and ALT was noticed on the 91st day at 3000 mg/kg BW (Figures 5(a) and 5(b)) while ALP activity increased significantly at 3000 mg/kg on the 61st and 91st days and at 1500 mg/kg at 91st day (Figure 5(c)). Liver histology revealed vacuolar degeneration at 3000 mg/kg BW on the 91st day (Figure 6(c)). This was however reversed during the posttreatment period (Figure 6(d)). No histological changes were observed at 1500 mg/kg BW (Figure 6(b)) and in the negative control (Figure 6(a)).

## 4. Discussion 

In this study,* G. latifolium *fruit extract and fractions generally showed antioxidant and immunomodulatory activities. The ethanol fruit extract was also found to be safe after 90-day toxicity study.

The ethanol extract, ethyl acetate, and methanol fractions diminished the deep purple colour of DPPH at 517 nm, an indication of their antioxidant potentials. This could be due to the phytochemical constituents of the extract. Flavonoids [24] and saponins [25] have been shown to possess electron and hydrogen donating properties, respectively.

Exposure of the animals to* G. latifolium *fruit extract, ethyl acetate, and methanol fractions increased the activities of catalase (CAT), superoxide dismutase (SOD) enzymes, and decreased malondialdehyde (MDA). SOD converts superoxide radicals to hydrogen peroxide, while CAT converts the hydrogen peroxide to water and oxygen [1]. Reactive oxygen species degrade polyunsaturated lipids forming malondialdehyde [26]. Therefore, increasing the activities of these enzymes results in increased antioxidant activities and ability to reduce lipid peroxidation. In 2003, Ugochukwu and coworkers [6, 8] using Wistar rats reported SOD and glutathione peroxidase activities and decreased MDA levels. Also, in 2006, Nwanjo and coworkers [27] reported increased antilipid peroxidase and SOD effects. Their assessments however were based on the use of the ethanol leaf extract, not the fruit. These effects could be due to the phytoconstituents of the extracts.

Phenolic compounds have been noted to induce the activation of multidrug resistant protein gene [28]. This property of phenolic compounds may have resulted in diminished liver uptake of CCL_4_ leading to reduced free radical formation and cellular damage. The potent antioxidant activity of flavonoids, their ability to scavenge hydroxyl radicals, may be the most important function of flavonoids and underlies many of their actions in the body [29]. They do not only have direct antioxidant activity but also have sparing effects on other antioxidant compounds [30]. Their capacity to modify membrane dependent process, such as free radical induced membrane lipid peroxidation, is related not only to their structural characteristics but also to their ability to interact with and penetrate the lipid bilayers [31]. Saponins have been documented to produce significant increase in both catalase and SOD activity in the heart with significant decrease in malonyldialdehyde [32]. Tannin extract from* G. latifolium* leaves has been found to inhibit food deterioration through inhibition of oxidative enzymes such as lipoxygenase [33]. Following the diverse mechanisms of antioxidant activities of saponins, flavonoids, tannins, and terpenoids [34], it is most likely that the antioxidant activity exhibited by the extract and fractions of* G. latifolium* fruit may be mediated by more than a single mechanism.

The extract and fractions significantly (*P* < 0.05) enhanced cellular immunity (increased phagocytic index), increased delayed hypersensitivity reaction (increased SRBC induced hypersensitivity reaction), increased innate immunity (increase in leukocyte count), and increased stimulation of the mouse IgG_1_ and IgG_2a_. These indicate that extracts of* G. latifolium* can boost immunity. Free radicals weaken the immune system [35]; hence the antioxidant effect of extract and fractions could be linked to its immunostimulant activities. This observation is supported by the report that supplementation with antioxidants has been found to protect immune response from certain environmental sources of free radicals [2, 36].

The acute toxicity studies revealed that the extract did not cause any death in both mice and rats up to 5000 mg/kg. The nonsignificant (*P* > 0.05) reduction of PCV, Hb concentration, and RBC count is an indication of unlikeliness of the extract to cause anaemia [21].

AST, ALT, and ALP are liver marker enzymes. Their increase in serum is roughly proportional to the extent of liver damage [7]. The significant (*P* < 0.05) elevation of ALT, AST, and ALP was noticeable at higher doses. Histological examination of the liver showed that the extract at 3000 mg/kg produced vacuolar degeneration at the 91st day which however showed convincing signs of reversibility after 28-day posttreatment. Vacuolar changes usually occur due to overaccumulation of its storage products or waste products and can also occur secondarily due to endocrine disorder or increased intake of steroids [37]. This effect could have occurred due to the high concentration of the extract (3000 mg/kg) or due to its high steroid content that may have led to glycogen accumulation. Glycogen accumulation has been documented to be a leading cause of vacuolar degeneration in dogs [37].

Our findings confirm the value of* G. latifolium *fruit extract as an antioxidant and immunostimulator in rats. Although we are much encouraged by these effects of the extract in our rat model, further study of the mutagenic and teratogenic effects is needed before the use of* G. latifolium *fruit extract in humans is justified.

## 5. Conclusion

The extracts and fractions of* G. latifolium* fruit have antioxidant and immunostimulant effects. This justifies the folkloric use in general body healing and maintenance of healthy living. Its long term administration is safe especially at lower doses. However, its liver toxicity at high dose is reversible.

## Figures and Tables

**Table 1:** Phytochemical analysis and yield of *G.  latifolium* ethanol fruit extract and fractions.

Phytocompounds	Ethanol extract	*n*-Hexane fraction	Chloroform fraction	Ethyl acetate fraction	Methanol fraction
Alkaloids	+++	++	++	−	
Saponins	+++	−	−	−	+++
Flavonoids	++++	−	−	+++	+++
Glycosides	+++	−	−	−	+++
Terpenoids	+++	−	++	−	−
Steroids	++++	+++	++	−	−
Fats and oil	++	++	−	−	−
Carbohydrate	++	−	−	−	++
Proteins	+++	−	−	++	+++
Resins	+++	+++	++	−	−
Tannins	++	−	−	−	++
Yield (%)	28.6^a^	4.2^b^	22.5^b^	26.3^b^	43.8^b^

−, not present; +, present in small concentration; ++, present in moderately high concentration; +++, present in high concentration; ++++, abundantly present.

^a^Calculated from 1000 g powdered fruit; ^b^Calculated from 116 g extract.

**Table 2:** Effect of extract and fractions of *G. latifolium *fruit on serum catalase and superoxide dismutase activity.

Treatment	Dose (mg/kg)	Catalase (U/L)		Superoxide dismutase (U/L)	
		Pretreatment	Posttreatment	Pretreatment	Posttreatment
Ethanol extract	200	1.8 ± 0.4	2.5 ± 0.6	11.9 ± 0.5	^*^22.0 ± 0.8
	400	2.0 ± 0.5	^*^2.8 ± 0.8	11.8 ± 0.4	^*^22.3 ± 0.6
Ethyl acetate fraction	200	2.1 ± 0.6	^*^2.9 ± 0.4	11.7 ± 0.5	^*^22.4 ± 0.9
	400	1.9 ± 0.5	^*^3.1 ± 0.7	11.7 ± 0.3	^*^24.8 ± 0.7
Methanol fraction	200	2.0 ± 0.4	^*^3.1 ± 0.6	11.8 ± 0.5	^*^23.3 ± 0.8
	400	2.1 ± 0.5	^*^3.3 ± 0.7	11.9 ± 0.4	^*^25.5 ± 0.9
Vitamin C	200	2.0 ± 0.4	^*^2.9 ± 0.6	11.8 ± 0.5	^*^23.4 ± 0.8
10% tween 80	10 mL/kg	2.0 ± 0.4	1.5 ± 0.8	11.7 ± 0.5	9.3 ± 0.6

*N* = 5, ^*^ *P* < 0.05.

**Table 3:** Effect of the extract and fractions on carbon clearance and delayed type hypersensitivity reaction (DTHR).

Treatment	Dose (mg/kg)	Phagocytic index	DTHR response
Ethanol extract	200	0.012 ± 0.003	^*^0.10 ± 0.008
	400	^*^0.020 ± 0.002	^*^0.11 ± 0.005
Ethyl acetate fraction	200	^*^0.021 ± 0.001	^*^0.10 ± 0.006
	400	^*^0.043 ± 0.004	^*^0.11 ± 0.004
Methanol fraction	200	^*^0.025 ± 0.003	^*^0.11 ± 0.004
	400	^*^0.052 ± 0.003	^*^0.13 ± 0.006
Noni capsule	50	^*^0.061 ± 0.005	^*^0.17 ± 0.008
Normal saline	10 mL/kg	0.010 ± 0.002	0.05 ± 0.001

*N* = 5, ^*^ *P *< 0.05.

**Table 4:** Effect of the extract and fractions on total and differential leukocyte counts.

Treatment	Dose (mg/kg)	TLC (mm^3^)	DLC (%)	
			Neutrophil	Lymphocyte
Ethanol extract	200	4600 ± 21	34.75 ± 1.2	65.25 ± 3.3
	400	^*^4725 ± 26	23.00 ± 1.4	75.50 ± 5.0
Ethyl acetate fraction	200	^*^4900 ± 23	29.75 ± 1.0	70.13 ± 3.1
	400	^*^5850 ± 28	^*^40.75 ± 1.6	59.25 ± 2.8
Methanol fraction	200	^*^4900 ± 20	23.25 ± 1.4	75.25 ± 2.2
	400	^*^5325 ± 22	19.50 ± 1.1	79.50 ± 2.6
Noni capsule	50	^*^7150 ± 24	20.25 ± 1.2	79.65 ± 1.4
Normal saline	10 mL/kg	4550 ± 19	32.00 ± 1.3	67.75 ± 1.7

*N* = 5, ^*^ *P* < 0.05.

**Table 5:** Haemagglutination antibody titer: primary antibody response.

Treatment	Dose (mg/kg)	Ovalbumin antigen		Tetanus toxoid antigen	
		IgG_1_	IgG_2a_	IgG_1_	IgG_2a_
Ethanol extract	200	^*^0.33 ± 0.006	^*^0.13 ± 0.004	^*^0.14 ± 0.006	^*^0.08 ± 0.003
	400	^*^0.30 ± 0.004	^*^0.18 ± 0.010	^*^0.15 ± 0.008	^*^0.14 ± 0.006
Ethyl acetate fraction	200	^*^0.17 ± 0.003	^*^0.10 ± 0.002	^*^0.12 ± 0.005	^*^0.10 ± 0.003
	400	^*^0.19 ± 0.006	^*^0.12 ± 0.004	^*^0.15 ± 0.007	^*^0.11 ± 0.005
Methanol fraction	200	^*^0.19 ± 0.011	^*^0.10 ± 0.003	^*^0.14 ± 0.005	^*^0.12 ± 0.004
	400	^*^0.21 ± 0.020	^*^0.14 ± 0.015	^*^0.17 ± 0.009	^*^0.16 ± 0.007
Noni capsule	50	^*^0.62 ± 0.026	^*^0.52 ± 0.039	^*^0.56 ± 0.021	^*^0.43 ± 0.026
Normal saline	10 mL/kg	0.05 ± 0.002	0.04 ± 0.007	0.04 ± 0.002	0.03 ± 0.002

*N* = 5, ^*^ *P* < 0.01.

**Table 6:** Haemagglutination antibody titer: secondary antibody response.

Treatment	Dose (mg/kg)	Ovalbumin antigen		Tetanus toxoid antigen	
		IgG_1_	lgG_2a_	lgG_1_	LgG_2a_
Ethanol extract	200	^*^0.47 ± 0.017	^*^0.21 ± 0.035	^*^0.11 ± 0.015	^*^0.11 ± 0.012
	400	^*^0.46 ± 0.014	^*^0.21 ± 0.023	^*^0.16 ± 0.018	^*^0.23 ± 0.014
Ethyl acetate fraction	200	^*^0.49 ± 0.017	^*^0.15 ± 0.012	^*^0.15 ± 0.013	^*^0.17 ± 0.010
	400	^*^0.34 ± 0.013	^*^0.14 ± 0.010	^*^0.15 ± 0.011	^*^0.15 ± 0.009
Methanol fraction	200	^*^0.50 ± 0.013	^*^0.19 ± 0.013	^*^0.15 ± 0.017	^*^0.19 ± 0.011
	400	^*^0.30 ± 0.010	^*^0.17 ± 0.014	^*^0.16 ± 0.018	^*^0.12 ± 0.019
Noni capsule	50	^*^1.10 ± 0.036	^*^0.99 ± 0.045	^*^0.86 ± 0.037	^*^0.73 ± 0.013
Normal saline	10 mL/kg	0.08 ± 0.001	0.05 ± 0.007	0.04 ± 0.006	0.03 ± 0.005

*N* = 5, ^*^ *P* < 0.01.
